# Preparation of CPD Photolyase Nanoliposomes Derived from Antarctic Microalgae and Their Effect on UVB-Induced Skin Damage in Mice

**DOI:** 10.3390/ijms232315148

**Published:** 2022-12-02

**Authors:** Changfeng Qu, Nianxu Li, Tianlong Liu, Yingying He, Jinlai Miao

**Affiliations:** 1 Key Laboratory of Marine Eco-Environmental Science and Technology, First Institute of Oceanography, Ministry of Natural Resources, Qingdao 266061, China; 2 Laboratory for Marine Drugs and Bioproducts, Qingdao Pilot National Laboratory for Marine Science and Technology, Qingdao 266237, China; 3 Marine Natural Products Research and Development Laboratory, Qingdao Key Laboratory, Qingdao 266061, China

**Keywords:** Antarctic microalgae, CPD photolyase, nanoliposome, DNA repair, MAPK pathway, MMP

## Abstract

UVB radiation is known to trigger the block of DNA replication and transcription by forming cyclobutane pyrimidine dimer (CPD), which results in severe skin damage. CPD photolyase, a kind of DNA repair enzyme, can efficiently repair CPDs that are absent in humans and mice. Although exogenous CPD photolyases have beneficial effects on skin diseases, the mechanisms of CPD photolyases on the skin remain unknown. Here, this study prepared CPD photolyase nanoliposomes (CPDNL) from Antarctic *Chlamydomonas* sp. ICE-L, which thrives in harsh, high-UVB conditions, and evaluated their protective mechanisms against UVB-induced damage in mice. CPDNL were optimized using response surface methodology, characterized by a mean particle size of 105.5 nm, with an encapsulation efficiency of 63.3%. Topical application of CPDNL prevented UVB-induced erythema, epidermal thickness, and wrinkles in mice. CPDNL mitigated UVB-induced DNA damage by significantly decreasing the CPD concentration. CPDNL exhibited antioxidant properties as they reduced the production of reactive oxygen species (ROS) and malondialdehyde. Through activation of the NF-κB pathway, CPDNL reduced the expression of pro-inflammatory cytokines including IL-6, TNF-α, and COX-2. Furthermore, CPDNL suppressed the MAPK signaling activation by downregulating the mRNA and protein expression of ERK, JNK, and p38 as well as AP-1. The MMP-1 and MMP-2 expressions were also remarkably decreased, which inhibited the collagen degradation. Therefore, we concluded that CPDNL exerted DNA repair, antioxidant, anti-inflammation, and anti-wrinkle properties as well as collagen protection via regulation of the NF-κB/MAPK/MMP signaling pathways in UVB-induced mice, demonstrating that Antarctic CPD photolyases have the potential for skincare products against UVB and photoaging.

## 1. Introduction

Ultraviolet-B (UVB) radiation (with a wavelength of 280–320 nm) from sunlight induces DNA damage though the dimerization of the two adjacent pyrimidine bases, triggering *cis-syn* cyclobutane pyrimidine dimer (CPD) and pyrimidine-pyrimidone (6-4) photoproduct (6-4 PP) [1,2], which potentially can lead to mutations and cell death by blocking DNA replication and transcription [3]. If the DNA lesions are not repaired, they can subsequently cause photoaging and skin cancers such as basal cell carcinoma, squamous cell carcinoma, and melanomas, the incidence of which has risen substantially [4,5]. In mammalian cells, CPDs are the major lesions, accounting for at least 80% of the total UVB-induced photoproducts and being more mutagenic than others [6]. CPDs have also been predominantly attributed to DNA lesions in inducing sunlight-dependent skin cancer mutations; for example, cytosine-deaminated CPDs have been found to be the leading premutagenic lesions responsible for mutations in melanomas [7].

Mammalian cells (including human) are able to repair UVB-induced DNA lesions through nucleotide excision repair (NER), and this pathway is the only DNA-repair pathway to remove UVB-induced photoproducts in placental mammals [8,9]. However, the NER pathway is complex and multifaceted, involving numerous of proteins and intermediate steps [10]. However, in other organisms there is an additional direct repair pathway, which rapidly repair DNA lesions by catalyzing the split dimers back to the original undamaged bases in a light-dependent process, called DNA photolyases [11,12]. These photolyases show substrate specificity for either CPD photolyases (CPDs) or 6-4 photolyases (6-4 PP), meaning that CPD photolyases can repair CPDs but cannot repair 6-4 PP and vice versa [13]. Furthermore, CPD photolyases have been confirmed to prevent the UVB-induced apoptosis in NER-proficient human cells, while 6-4 photolyases have no effects [10]. Remarkably, both photolyases are absent in placental mammals (including humans and mice) [14,15]. CPD photolyases have been found in bacteria, fungi, plants, invertebrates, vertebrates, and marsupial mammals [14,16,17,18,19,20]. Previous findings indicated humans and mice do not have the genes encoding CPD photolyases [15]; moreover, as a macromolecule, CPD photolyases do not penetrate the skin directly. Loading CPD photolyases into a variety of liposomes, which causes fluidization of the cell membrane and encapsulates the bioactive enzymes to pass through the skin, is one of the most effective strategies [21,22].

Application of exogenous CPD photolyases has been demonstrated to prevent existing DNA damage both in vivo and in vitro [23,24,25,26,27,28,29,30,31]. CPD photolyase liposomes have been used in cosmetic and pharmaceutical preparations to reduce photoaging and UV radiation skin damage [32,33,34]. Liposomes of CPD photolyase applied to UVB-induced human skin decreased epidermal CPD and prevented immunosuppressive effects by restoring keratinocyte intercellular adhesion molecule-1 expression [25]. Clinical studies also showed that the addition of CPD photolyases to conventional sunscreens significantly reduced CPDs formation, free radical-induced protein damage, and apoptotic cell death compared to conventional sunscreen alone [32,33]. Hence, the combination of CPD photolyases and conventional sunscreen has been used as an adjuvant therapy in patients with actinic keratosis (AK), non-melanoma skin cancer (NMSC), and xeroderma pigmentosum (XP), improving visible and cancerous lesions and protecting against the appearance of new lesions [28,35,36,37,38]. However, sunscreens incorporating CPD photolyases have not been widely used due to the relatively high cost and lack of stability of the protein extracts. Therefore, efficient and fast CPD photolyases have yet to be explored. Moreover, despite the application of CPD photolyases, their mechanisms on skin, particularly with regard to UVB protection, are limited.

Antarctic microalgae frequently thrive in the extreme UVB radiation resulting from the thinning of the ozone layer over Antarctica [39]. The *Chlamydomonas* sp. ICE-L is a crucial component of Antarctic communities, possessing an efficient DNA photolyase to repair UVB-induced DNA lesions [19,40], which is essential for its survival under high-UVB circumstances. In the present study, CPD photolyase nanoliposomes (CPDNL) from the Antarctic *Chlamydomonas* sp. ICE-L were prepared. The optimal conditions for CPDNL preparations as well as CPDNL’s properties were evaluated. More importantly, we estimated the protective effects of CPDNL on UVB-induced skin damage in mice. To further explore the potential mechanisms, the CPDs’ formation, oxidative stress, inflammation cytokines, and molecular pathways were investigated. For the first time, this study evaluated the possible mechanisms for the effects of CPDNL from Antarctic microalgae against UVB-induced skin damage.

## 2. Results

### 2.1. Characterization of CPDNL

#### 2.1.1. Expression and Purification of Recombinant CPD Photolyase Protein

**Figure 1:**
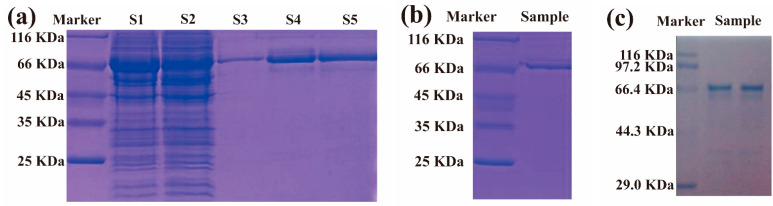
The SDS-PAGE and Western blot analysis of CPD photolyase protein. (**a**) SDS-PAGE analysis during Ni-NTA affinity chromatographic purification process (S1: sample loading; S2: effluent; S3–S5: fraction with gradient elution); (**b**) SDS-PAGE analysis of purified CPD photolyase protein; (**c**) Western blot analysis of purified CPD photolyase protein.

Sodium dodecyl sulfate polyacrylamide gel electrophoresis (SDS-PAGE) analysis showed that the CPD photolyases were successfully expressed in the bacterial strain Transetta (DE3)-pEASY^®^-Blunt E2 (Figure 1a). After the optimization of the recombinant bacterial culture and protein-overexpression-induced conditions, the CPD photolyases were purified by the Ni-NTA affinity chromatographic column, and the elution fraction (S3, S4, and S5) with a molecular weight of approximately 66 kDa was the target CPD photolyase protein. Then, the CPD photolyase protein quantity in recombinant *Escherichia coli* was estimated to be 1755–1884 μg/g (wet weight of bacteria). Purified CPD photolyase protein was affirmed by SDS-PAGE and Western blot analysis (Figure 1b,c).

#### 2.1.2. Optimization of CPDNL Preparation Conditions 

The RSM experimental design and results are shown in Table 1. The quadratic polynomial regression equation using ANOVA is generated as follows:

Y1 (Particle Size) = + 98 − 36.83A − 38.92B + 10.17C − 34.75D + 18AB − 12.5AC + 7.5AD − 35.75BC + 22.5BD + 4.75CD + 18.25A² + 25.12B² + 38.75C² + 8.37D²

Y2 (Encapsulation Efficiency) = + 70.88 − 12.16A − 4.93B − 3.01C − 6.84D + 3.88AB − 0.1AC + 3.6AD + 2.2BC − 0.5BD + 0.575CD − 14.32A² − 11.54B² − 4.82C² − 4.59D²

The ANOVA results of the quadratic model were given in Table 2. For both Y1 and Y2, the F-value of 4.39 and 2.50, respectively, implied that the model was significant; furthermore, the significance of the corresponding coefficients (*p*-value = 0.0045 for Y1; *p*-value = 0.0488 for Y2) indicated that the model was suitable for this experiment. The lack of fit was more than 0.1 (*p*-value = 0.1113 for Y1; *p*-value = 0.1006 for Y2), suggesting the value was not significant relative to the pure error.

The linear term of lecithin to cholesterol ratio (A), with *p* = 0.0022; CPD photolyases to lecithin (B), with *p* = 0.0015; and sonication time (D), with *p* = 0.0034, had a highly significant effect on particle size. The quadratic term of oil to water ratio (C^2^), with *p* = 0.012, had a significant effect.

For encapsulation efficiency, the effect of the linear term of the lecithin to cholesterol ratio (A, *p* = 0.0028) and the quadratic term of the lecithin to cholesterol ratio (A2, *p* = 0.0073) was highly significant, and the effect of the quadratic term of the CPD photolyases to lecithin ratio (B2, *p* = 0.0240) was significant.

The optimum conditions were 2.5 for the lecithin to cholesterol ratio, 0.15 for the CPD photolyases to lecithin, 2.1 for the oil to water ratio, and 9.7 min for the sonication time; accordingly, the optimized CPDNL preparation process was designed. The predicted values of particle size and entrapment efficiency were 108.8 nm and 70.5%, respectively.

#### 2.1.3. Properties of the Optimized CPDNL

**Figure 2:**
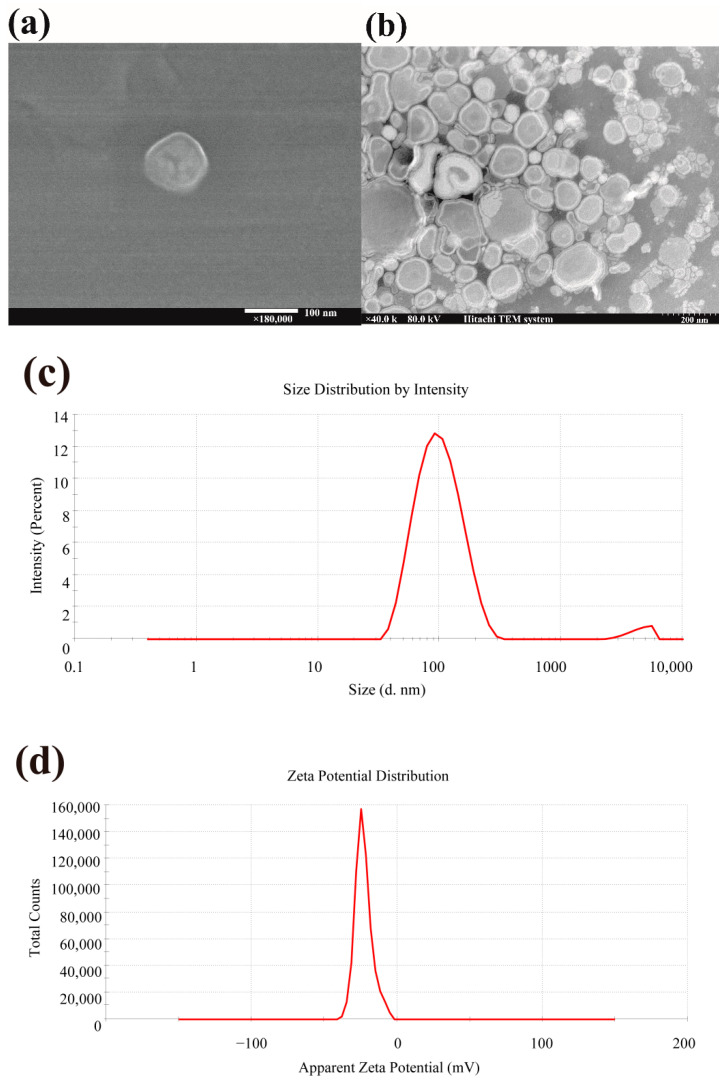
Characterization of CPD photolyase nanoliposomes. (**a**) Scanning electron microscope (SEM) image; (**b**) transmission electron microscope (TEM) image; (**c**) particle size distribution; (**d**) zeta potential.

The results of the scanning electron microscope (SEM) and transmission electron microscope (TEM) (Figure 2) illustrated that the optimized CPDNL presented spherical vesicles and exhibited a bilayer structure. The particle size distribution of CPDNL was 105.5 ± 6.3 nm, which was close to the predicted values of 108.8 nm. The polydispersity index (PDI) and zeta potential of CPDNL were 0.28 ± 0.001 and negative 21.7 ± 1.9 mV, respectively, indicating that the CPDNL had a steady state and good dispersion. The entrapment efficiency was obtained as 63.3 ± 2.1%.

#### 2.1.4. Enzyme Activity of CPDNL

**Figure 3:**
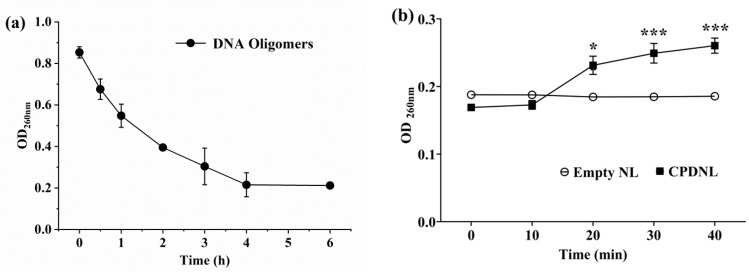
Enzyme activity of CPDNL. (**a**) Absorption at 260 nm during the generation of CPD substrates; (**b**) absorption at 260 nm after the addition of empty NL and CPDNL. Empty NL: nanoliposomes without CPD photolyases; CPDNL: nanoliposomes of CPD photolyases. Data represent means ± SEM. * *p* < 0.05 and *** *p* < 0.001 compared to empty NL.

The decrease in absorbance for the OD_260_ of the DNA oligomers indicated the CPD substrates were generated by UVB radiation (Figure 3a). After the addition of nanoliposomes, the OD_260_ of the empty NL remained unchanged at a low value of 0.186 ± 0.002, whereas the OD_260_ of the CPDNL gradually increased, reaching the maximum of 0.260 ± 0.025 at 40 min (Figure 3b). Moreover, the OD_260_ of the CPDNL was significantly higher than that of the empty NL from 20 min to 40 min. The results demonstrated that the CPD substrates were repaired by CPDNL, and the empty NL had no repair activity by the CPDs. These results also confirmed that the CPD photolyases were entrapped in nanoliposomes.

### 2.2. Protective Effects of CPDNL on Skin Damage Induced by UVB Radiation

#### 2.2.1. Effects of CPDNL on the External Appearance and Histopathology of Skin

**Figure 4:**
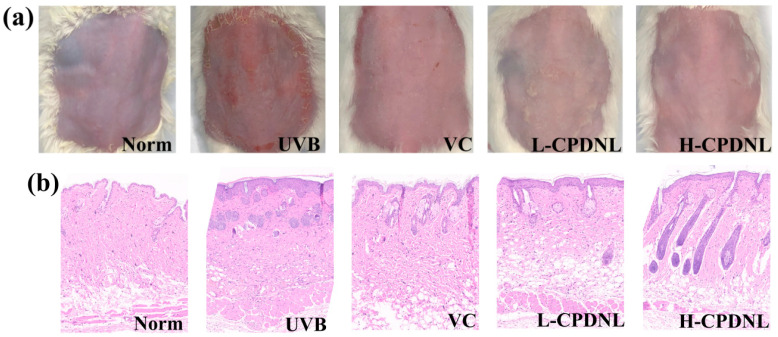
The external appearance and histopathology of skin tissues. (**a**) Images for the dorsal skin of mice; (**b**) hematoxylin and eosin (H&E) pathological images of skin. VC: vitamin C group; L-CPDNL: low-dose nanoliposomes of CPD photolyases; H-CPDNL: high-dose nanoliposomes of CPD photolyases.

UVB radiation obviously damaged the dorsal skin of mice and significantly caused erythema, wrinkles, and even scarring on the skin (Figure 4a). CPDNL and VC repaired the UVB damage, while CPDNL exhibited an obvious decrease in UVB-induced erythema compared with the UVB group. Histology photographs revealed that the Norm groups exhibited a healthy cellular structure with no morphological damage, including a thin epidermis, undamaged dermis, and neat collagen fibers (Figure 4b). UVB radiation caused a variety of lesions in skin tissues in the UVB groups, including hyperplasia, edema, cellular sunburn, and cell apoptosis. Although the histological changes of the skin tissue in the VC groups were improved, there was still the appearance of wrinkles. Compared to the UVB groups, the thickness of the epidermis and the amorphous material at the epidermal–dermal junction were significantly decreased in both CPDNL groups, the dermis appeared complete and neat, and the disorganized collagen fibers and the infiltrated inflammatory cells were reduced.

#### 2.2.2. CPDNL Reduced CPD Formation

**Figure 5:**
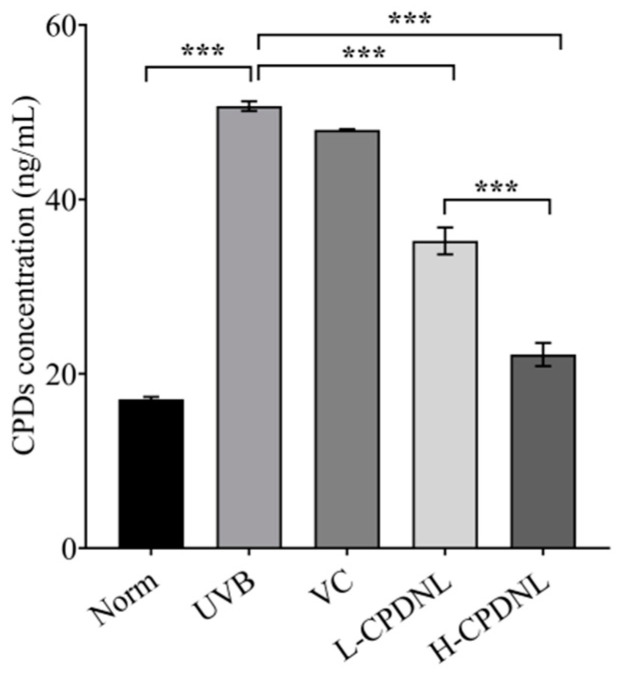
The accumulation of cyclobutane pyrimidine dimers (CPDs, ng/mL). Data represent means ± SEM. *** indicates *p* < 0.001.

As shown in Figure 5, UVB radiation significantly increased the CPDs, reflecting the low-efficient NER self-repair in mice skin. Both low and high doses of CPDNL significantly decreased the CPD concentration in comparison to UVB groups, from 50.70 ng/mL to 35.24 ng/mL and 22.22 ng/mL, respectively. The detectable concentration of the CPDs in the H-CPDNL groups was 56.2% lower than that in the UVB groups. The CPDs of high-dose CPDNL were significantly lower than those of low-dose CPDNL. There was no significant difference between the VC and UVB groups, showing that CPDNL but not VC efficiently repaired CPD photoproducts after UVB radiation.

#### 2.2.3. CPDNL Enhanced Hydroxyproline Content

**Figure 6:**
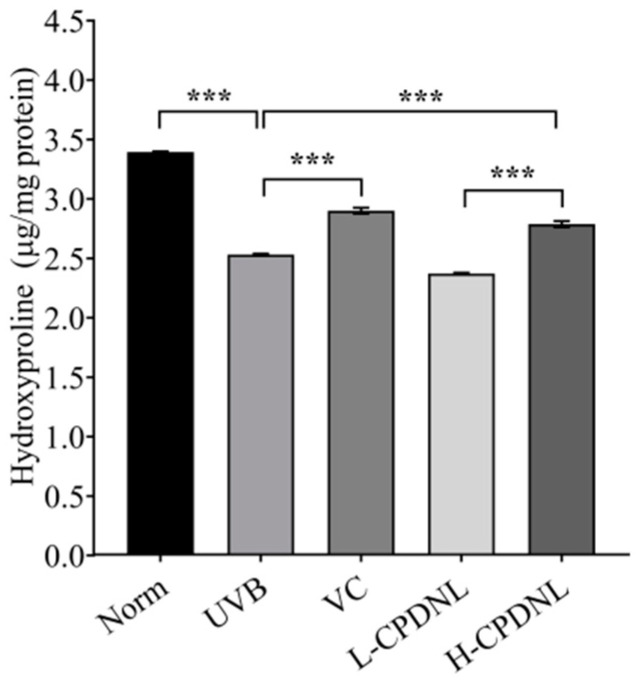
The hydroxyproline content (μg/mg protein) of skin tissue. Data represent means ± SEM. *** indicates *p* < 0.001.

Hydroxyproline is the major component of collagen and establishes and maintains collagen structure, so it is traditionally used as an indicator to quantify collagen [41]. The content of hydroxyproline in mice skin was significantly reduced after UVB exposure (Figure 6). The reduction in hydroxyproline led to the appearance of wrinkles and photoaging. High-dose CPDNL significantly increased the hydroxyproline content of the UVB groups by 10.3%, while there was no significant difference between the L-CPDNL and UVB groups. The hydroxyproline content of the high-dose CPDNL was significantly higher than that of the low-dose CPDNL, indicating that the effect of high-dose CPDNL on collagen protection was better.

#### 2.2.4. CPDNL Ameliorated Oxidative Stress Level

**Figure 7:**
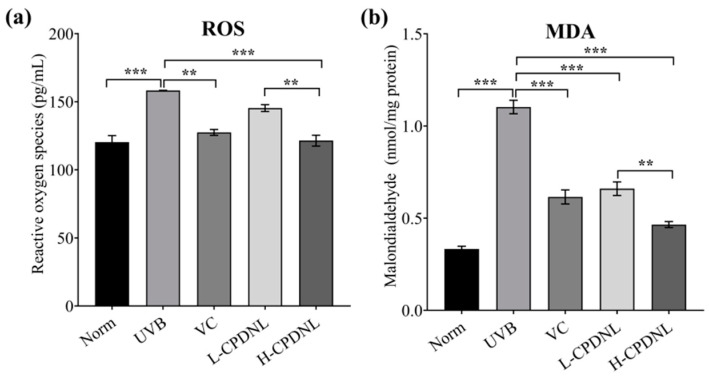
The oxidative stress levels. (**a**) Reactive oxygen species (ROS) production (pg/mL); (**b**) malondialdehyde (MDA) concentration (nmol/mg protein). Data represent means ± SEM. ** *p* < 0.01 and *** *p* < 0.001.

Reactive oxygen species (ROS) are known to be an effective biomarker to assess oxidative stress in the photoaging process [42]. UVB radiation induced a significant increase in ROS production, suggesting that the antioxidant capacity of the skin was weakened (Figure 7). The higher production of ROS in skin tissue may be due to the higher cellularity or inflammation induced by UVB radiation. The high dose of CPDNL group as well as the VC groups markedly suppressed ROS production, and the ROS in H-CPDNL returned to the Norm level. The ROS production of L-CPDNL was also found to be slightly lower than that of the UVB groups, but without statistical significance. The malondialdehyde (MDA) content also reflected the degree of lipid peroxidation. UVB radiation significantly increased the MDA production in skin tissues. Compared to the UVB groups, both the low- and high-dose CPDNL groups as well as the VC groups significantly reduced the MDA content by 40.2%, 57.8%, and 44.2%, respectively. Overall, the effect of high-dose CPDNL on anti-oxidative activity is better than that of low-dose CPDNL.

#### 2.2.5. CPDNL Attenuated Inflammation

**Figure 8:**
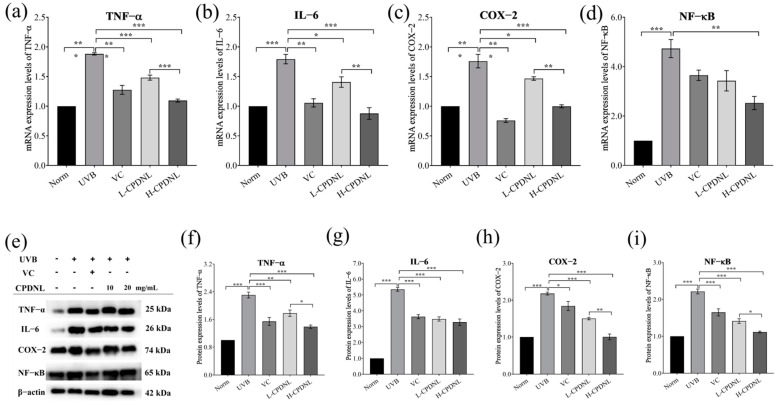
The expression of inflammation cytokines. The mRNA expression of TNF-α (**a**), IL-6 (**b**), COX-2 (**c**), and NF-κB (**d**) in skin tissue was detected. The protein expression level of inflammation factors (**e**) including TNF-α (**f**), IL-6 (**g**), COX-2 (**h**), and NF-κB (**i**) was determined by Western blot analysis. Data are presented as mean ± SEM. * *p* < 0.05, ** *p* < 0.01, and *** *p* < 0.001.

The increase in ROS induced by UVB promoted skin inflammation [43]. Next, we evaluated the influence of CPDNL on inflammation in UVB-induced mice skin, which is regulated by pro-inflammatory cytokines. UVB-induced groups increased the gene and protein expression of TNF-α, IL-6, COX-2, and NF-κB (Figure 8). The high-dose CPDNL groups significantly downregulated the mRNA and protein expression of pro-inflammatory TNF-α, IL-6, COX-2, and NF-κB. Except for NF-κB, which had no apparent differences, the L-CPDNL groups significantly reduced the mRNA expression of TNF-α, IL-6, and COX-2. Similarly, the protein expression of those cytokines in the low- and high-dose CPDNL groups and the VC groups had a significant difference compared to the UVB groups, and the effect of CPDNL was dose-dependent. The observations indicated that CPDNL could regulate inflammatory cytokines and possess anti-inflammatory properties.

#### 2.2.6. CPDNL Inhibited MMPs via the Suppression of MAPK/AP-1 Signaling Pathways

**Figure 9:**
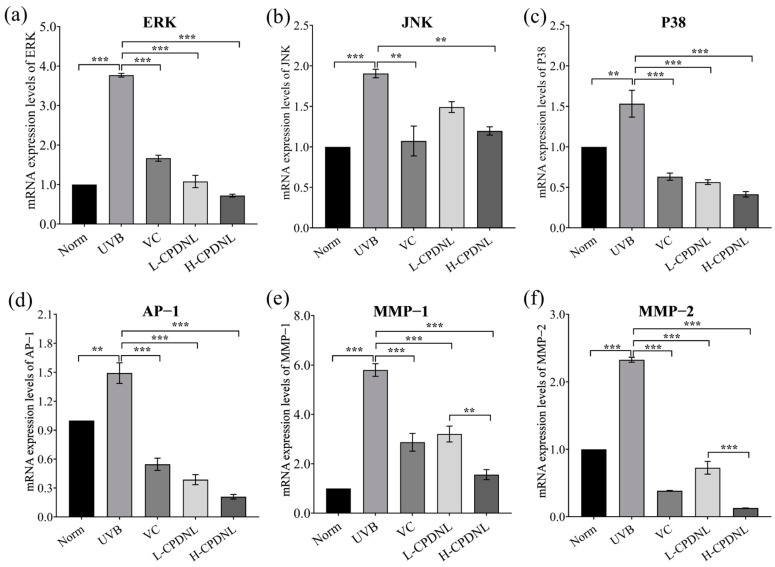
The effect of CPDNL on mRNA expression of MAPK/AP-1/MMP signaling pathways in UVB-induced skin. (**a–c**) The mRNA expression of ERK, JNK, and P38; (**d**) the mRNA expression of AP-1; (**e**,**f**) the mRNA expression of MMP-1 and MMP-2. Data are presented as mean ± SEM. ** *p* < 0.01 and *** *p* < 0.001.

**Figure 10:**
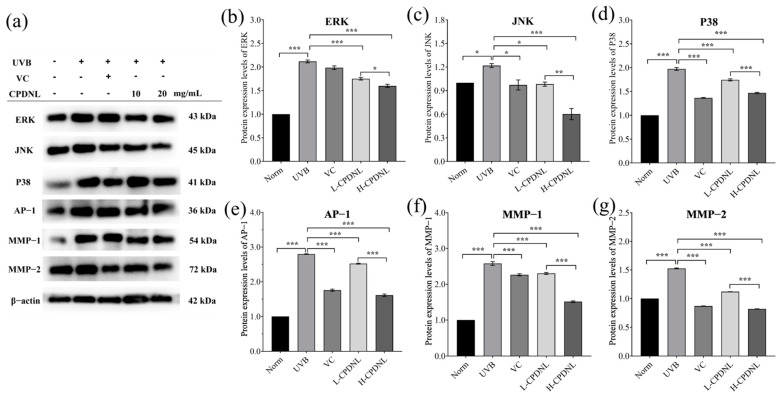
The effect of CPDNL on protein level of MAPK/AP-1/MMP signaling pathways in UVB-induced skin. The protein expression level (**a**) of ERK (**b**), JNK (**c**), P38 (**d**), AP-1 (**e**), MMP-1 (**f**), and MMP-2 (**g**) was determined by Western blot analysis. Data are presented as mean ± SEM. * *p* < 0.05, ** *p* < 0.01, and *** *p* < 0.001.

To further investigate the mechanisms underlying CPDNL protection against UVB-induced skin damage, the mRNA and protein expression of multiple signaling pathways were examined. MAPK (mitogen-activated protein kinases) pathways mediate cellular responses by regulating collagen and are key regulators of skin homeostasis and inflammatory cytokine expression [44,45,46,47]. As expected, UVB activated the MAPK pathway through significant upregulation of the levels of ERK (extracellular signal-regulated kinase), JNK (c-Jun-N-terminal kinase), and P38 in skin tissue (Figure 9 and Figure 10). The high dose of CPDNL group significantly reduced the mRNA and protein expression of ERK, JNK, and P38, suggesting the activation of the MAPK pathway was inhibited. With the exception of the JNK mRNA expression, which had no apparent differences, the L-CPDNL groups significantly reduced the mRNA and protein expression of ERK and P38. Both MAPK pathways and AP-1 pathways are required for the regulation of matrix metalloproteinase (MMP) expression [48]. Transcription factor AP-1 was also activated by UVB. The low- and high-dose CPDNL groups and the VC groups remarkably downregulated AP-1 levels. MMPs are involved in the degradation of major extracellular matrixes such as type I collagen [49,50,51]. Both MMP-1 (termed fibroblast collagenase) and MMP-2 (termed gelatinase-A) were significantly upregulated by UVB and significantly downregulated by CPDNL in a dose-dependent manner, suggesting that CPDNL suppressed collagen degradation through the inhibition of MMP pathways. Moreover, the inhibition of MMP-1 and MMP-2 was linked to the suppression of MAPK and AP-1. Therefore, CPDNL protected against UVB-induced skin damage by regulating the MAPK/AP-1/MMP pathways. 

## 3. Discussion

The CPD photolyases of *Chlamydomonas* sp. ICE-L contain FAD as the primary catalytic cofactor and are photoreducible by blue light, allowing CPDs to be completely repaired in less than 50 min [19]. Furthermore, CPDs are the major mutagenic DNA lesions introduced by UVB in humans and mice; however, CPD photolyases that can directly repair CPDs are generally not present, and CPDs are only repaired via the NER pathway [10,14,15]. Consequently, the CPD photolyases of Antarctic *Chlamydomonas* sp. ICE-L are an excellent material for repairing UVB-induced skin damage and photoaging [52]. In this study, CPD photolyases of Antarctic microalgae were successfully encapsulated into nanoliposomes for the first time by the thin-film evaporation method, and so far there have been no reports about the preparation of CPD photolyase liposomes. The encapsulation efficiency was 63.3%, displaying a high encapsulation efficiency, compared to other enzymes such as SOD liposomes, ranging from 8% to 59% [53]. More importantly, the findings of the enzymatic activity of CPDNL, which was significantly higher than that of empty nanoliposomes, confirmed that CPD photolyases were efficiently and stably encapsulated.

UVB exposure has multiple biological effects on the skin, including erythema, hyperplasia, hyperkeratosis, inflammation, metabolic changes, and sunburn cell formation [54]. From previously reported studies, only liposomes containing CPD photolyases from the cyanobacterium *Anacystis nidulans* are known to exert improvement effects against UVB-induced damage [31]. In this study, the application of CPDNL from the Antarctic *Chlamydomonas* sp. ICE-L mitigated the adverse effects on the skin of mice. Epidermis thickening and dermis swelling were attenuated in the CPDNL group compared to the UVB-induced group. Additionally, the expressed CPD photolyases transgene from a marsupial, *Potorous tridactylus*, could also reduce acute skin effects and the increased resistance to UVB radiation exposure [23,27,30]. Therefore, the effects of the Antarctic *Chlamydomonas* CPD photolyases expressed in placental cells, such as human keratinocytes cells, which are the primary acceptors of UVB radiation in human skin [29,55], will be investigated in the future.

The results are consistent with previous studies showing that UVB radiation causes the accumulation of CPDs, which are major contributors to cell apoptosis, acute skin effects, and molecular changes [1,6,7,10,24,27]. UVB-induced alterations of mitochondrial morphology, lipophagy, and hypermetabolic switch were CPD-dependent, while CPDs modulated mitochondrial biogenesis and substrate oxidation in human keratinocytes [25]. Furthermore, residual CPDs induced by chronic UVB radiation persisted in the DNA and led to a delay in cell replication, which increased genomic instability and might favor carcinogenesis [56]. In this present study, CPDNL significantly reduced the amount of CPDs induced by UVB, whereas the VC groups had little effects. Moreover, a high CPDNL dose reversed CPDs concentration to normal levels, indicating that CPDNL has a greater efficiency. Similarly, several studies have demonstrated that CPD photolyases can remove CPDs and successfully protect against UVB-induced damage both in vivo and in vitro. Topical application of CPD photolyases liposomes from *Anacystis nidulans* to UVB-irradiated skin removed 40–45% of CPDs [31]. CPD photolyases from the *Potorous tridactylus* expressed in transgenic mice, which efficiently removed CPDs in the epidermis and dermis, and showed greater resistance to the deleterious effects of UVB than the 6-4 photolyases [27]; these photolyases transfected into human keratinocytes by pseudouridine-modified mRNA could remove more than 60% of CPDs within 1 h and block the signaling pathways triggered by CPDs [23,25]. 

UVB radiation causes the generation of free radicals (e.g., intracellular ROS) in skin, leading to oxidative stress, inflammation, photoaging, and even cancer [42]. Our results showed that high-dose CPDNL suppressed UVB-induced intracellular ROS production in the skin. The release of ROS in skin is believed to activate and modulate signaling pathways that may be involved in the pathogenesis of skin disorders or dermatological diseases [57], such as the NF-kB pathway, activator protein-1 (AP-1) pathway, and mitogen-activated protein kinases (MAPK) pathway [58,59]. ROS also trigger the release of pro-inflammation cytokines, such as TNF-α, IL-1, and COX-2 [60]. In addition, oxidative stress drives the production of oxidation products, such as MDA. In this study, both doses of CPDNL significantly reduced the MDA levels, and the high dose was significantly better than the low dose. Thus, CPDNL inhibited the UVB-induced ROS, as well as prevented the formation of MDA, which could mediate or reverse cellular antioxidant systems to protect the skin against the oxidative damage induced by UVB.

Consistent with previous studies [60,61,62], UVB radiation stimulated the inflammatory process in skin, significantly increased the expression levels of pro-inflammatory cytokines, including TNF-α and IL-6, and also increased the expression of COX-2. These pro-inflammatory cytokines also stimulated ROS accumulation, which is in accordance with our findings. CPDNL suppressed UVB-induced inflammation by significantly reducing the mRNA and protein expression of TNF-α, IL-6, and COX-2; moreover, the high-dose CPDNL was significantly better than the low-dose CPDNL. Except for pro-inflammatory cytokines, UVB-induced local inflammatory changes such as hyperplasia, edema, and leukocyte infiltration were suppressed by topically applied CPDNL. Exposure to UVB also induced activation of NF-κB. A high dose of CPDNL could significantly decrease the expression of NF-κB, indicating that CPDNL was supposed to protect against UVB-induced damage through the NF-κB signaling pathway. Thus, this study confirmed that CPDNL exhibited anti-inflammatory effects by inhibiting the NF-кB signaling pathways and suppressing the expression of the pro-inflammatory cytokines such as TNF-α, IL-6, and COX-2 induced by UVB. The inhibition of the NF-κВ signaling pathway might be an important mechanism for CPDNL in treating UVB-induced skin damage.

Generally, UVB-induced ROS production and skin inflammation are regulated by the activation of MAPK pathways, which consist of ERK, JNK, and p38 and play central roles in cell proliferation, differentiation, repair, apoptosis, and so forth [63]. As expected, UVB-induced stress activated these enzymes [64], and UVB significantly elevated the expression of ERK, JNK, and p38 in skin in this study. The expression of ERK, JNK, and p38 was markedly suppressed by a high dose of CPDNL, implying that these enzymes could differentially engage in CPDNL-mediated pharmacological activities, and CPDNL suppressed MAPK activation. Another major regulator of the MAPK pathway is AP-1, which is composed of the Jun and Fos family proteins and plays an important role in the processes of cell proliferation, cell differentiation, and cell survival, as well as UVB-induced skin tumors [48]. CPDNL also significantly inhibited UVB-induced AP-1 activation. 

Both fibroblast collagenase (MMP-1) and gelatinase-A (MMP-2) can initiate the degradation of native collagen [50,65]. Thereby, the upregulation of MMP-1 and MMP-2 is mainly responsible for collagen damage. The mRNA and protein expression of MMP-1 and MMP-2 in skin was significantly enhanced after UVB exposure but significantly decreased in the CPDNL group in a dose-dependent manner. In accordance with our results, Dong et al. [66] have also found that DNA repair enzymes liposomes resulted in a reduction in MMP-1 mRNA and protein expression in both the epidermis and dermis of the skin. In addition, CPDNL increased the concentration of hydroxyproline in the skin, which also indicated CPDNL was advantageous in terms of collagen. Since collagen degradation led to wrinkling as a characteristic of photoaging, our results also explained that CPDNL reduced the extent of wrinkling through inhibition of the collagen-degradation pathways. Moreover, the NF-κB/MAPK/AP-1/MMP signaling pathways are involved in the CPDNL repair process of UVB-induced skin damage in a complicated and mutually dependent manner. 

There is additional evidence that UVB-induced MAPK enhanced the expression of MMPs in skin by inducing the AP-1, NF-κB, and inflammatory cytokines [67]. Consistently, CPDNL was found to inhibit the expression of MMP-1 and MMP-2 by the activation of the MAPK, AP-1, and NF-κB signaling pathways. On the other hand, UVB-induced ROS generation can coordinately regulate multiple signaling pathways, stimulating the MAPK pathway by activating the ERK, JNK, and p38 signaling pathways and promoting the expression of MMPs, while downregulating NF-κB and AP-1 signaling [58,68,69,70], which is consistent with our findings. Taken together, the mechanisms of CPDNL protection against UVB-induced skin damage were based on the combined regulatory effect of multiple transcription factors. CPDNL application suppressed the transcription of MMP-1 and MMP-2 via inhibition of the MAPK pathways, by decreasing the phosphorylation of ERK, JNK, and p38 as well as the downregulation of the NF-κB and AP-1 pathways. The finding suggested that CPDNL might have the potential to be preventive agents against UVB-induced skin damage, photoaging, or skin cancers.

## 4. Materials and Methods

### 4.1. DNA Photolayses Expression and Purification

The Antarctic sea ice microalgae *Chlamydomonas* sp. ICE-L was obtained from the floating ice near the Zhongshan Research Station of Antarctica (S 69°48′, E 77°48′) (An, 2013). The algae were cultured in sterilized Provasoli seawater medium, with a diurnal cycle of 12 h: 12 h light: dark (L:D) and a light intensity of 40 μmol photos m^−2^ s^−1^ at 5 ± 1 °C. The CPD photolyase protein from *Chlamydomonas* sp. ICE-L was expressed in *Escherichia coli* according to the previous studies, with some modifications [19]. Briefly, for the construction of the CPD photolyase expression vector, a full-length CPD photolyase was inserted into pEASY^®^-Blunt E2 (TransGen biotech, Beijing, China). The recombinant plasmids were transformed into Transetta (DE3) (TransGen biotech, Beijing, China). Recombinant bacteria containing the transformed plasmid were cultured in Luria broth (LB) with kanamycin at 37 °C and 200 rpm, and then the protein expression was induced with isopropyl-β-d-thiogalactopyranoside (IPTG). After the optimization of protein-induced conditions, the expression protein was purified using ultrafiltration membrane and Ni-NTA affinity chromatographic column (AKTA purifier UPC10, GE Healthcare, Uppsala, Sweden). Purified CPD photolyase protein was affirmed by sodium dodecyl sulfate polyacrylamide gel electrophoresis (SDS-PAGE) and Western blot. The anti-6×His rabbit polyclonal antibody (D110002, Sangon, Shanghai, China) and the HRP-conjugated goat anti-Rabbit IgG (D110058, Sangon, Shanghai, China) were used as the primary and secondary antibodies, respectively. The prepared CPD photolyase protein was dried by vacuum freeze-drying equipment and stored at −80 °C.

### 4.2. Preparation of CPD Photolyase Nanoliposomes

The CPDNL were prepared using thin-film evaporation method. In brief, some amounts of lecithin and cholesterol were dissolved in ether: chloroform (3:2). The mixture of photolyase and phosphate-buffered saline (PBS, pH = 7.4) was added and sonicated using ultrasound probe (JY88-IIN, SCIENTZ, Ningbo, China). The solution was transferred to a round-bottom flask and conducted to remove the solvent and form a thin film using a rotary evaporator (RE2000A, Heidolph, Schwabach, Germany) at 20 °C for 30 min. With the addition of Tween-80 and PBS, the film was hydrated using stirring and vortex mixer. Ultimately, the above solution was sonicated for different amount of time and filtrated through a 0.22 μm Millipore filter membrane. The prepared nanoliposomes were stored at 4 °C.

To obtain the optimal conditions of CPDNL preparation, a response surface methodology (RSM) experimental design was conducted. A Box–Behnken design (BBD) of four factors and three levels was employed. The ratio of lecithin to cholesterol (A, weight to weight), CPD photolyases to lecithin (B, weight to weight), oil to water (C, volume to volume), and sonication time (D, min) were selected as independent variables. Briefly, the values of variable A (lecithin:cholesterol) were adjusted at 1:1, 2.5:1, and 4:1, with the lecithin amounts of 0.15 g, 0.25 g, and 0.45 g, respectively, and the cholesterol levels ranging from 0.03 g to 0.45 g. The values of variable B (CPD photolyases:lecithin) were set to 0.066:1, 0.133:1, and 0.2:1, respectively; the amount of CPD photolyases powder was fixed at 0.03 g, and the amount of lecithin was set to 0.15 g, 0.25 g, and 0.45 g, respectively. The solution of ether and chloroform (3:2) was taken as the oil phase, and the water phase was assigned to the CPD photolyases aqueous solution. Variable C (oil:water) was given the values of 1:1, 3:1, and 5:1, respectively. The total volume of oil was 7 mL, 10 mL, and 15 mL, respectively, while 3 mL and 7 mL of water were used. Variable D (sonication time) was assigned to 5 min, 12.5 min, and 20 min, respectively. The mean particle size (Y1, nm) and encapsulation efficiency (Y2, %) of CPDNL were selected as dependent variables. The experimental results were analyzed by Design Expert 8.0.6. A series of 29 experimental runs were in Table 1. The prepared CPDNL was dried by vacuum freeze-drying equipment and stored at −80 °C for the next experiments.

### 4.3. Characterization of CPD Photolyase Nanoliposomes

Prepared CPDNL were diluted using ultrapure distilled water. Mean particle size, polydispersity index (PDI), and zeta potential were measured by a dynamic light scattering (DLS) system (Zetasizer ZS90, Malvern, Malvern, UK) at 25 °C. A scanning electron microscope (Regulus 8100, Hitachi, Tokyo, Japan) and transmission electron microscope (HT7700, Hitachi, Tokyo, Japan) were also used to examine morphology of nanoliposomes.

The prepared CPDNP (1 mL) and a demulsifier Triton X-100 (5 mL) were mixed and centrifuged by an ultracentrifuge (Centrifuge 5804, Eppendorf, Hamburg, Germany) for 15 min at 12,000 rpm. Then, 100 μL of supernatant and 5 mL of PBS were used to determine the total protein concentration through BCA Protein Assay (Boster, Wuhan, China), in accordance with the instructions of the manufacturer. A protein concentration of nanoliposomes without demulsifier was used as the non-encapsulated free protein. The encapsulation efficiency (EE, %) was calculated based on the protein loaded inside the nanoliposomes (the total protein concentration minus the non-encapsulated protein concentration) divided by the total protein concentration.

The activity of CPD photolyases was measured as in previous studies [19], with some modifications. The CPD substrates were prepared by irradiation of DNA oligomers (5′-TTT TTT TTT TTT TTT T-3′) using a UVB lamp (280–320 nm) on ice for 4 h. The assay system contained 1 μM CPD photolyases, 5 μM substrates, 1 mM DTT, and 0.5 mM FAD in repair buffer (50 mM Tris/Cl, 100 mM NaCl, 1 mM EDTA, 100 mg mL^−1^ BSA, 10% (*w*/*v*) glycerol, pH 7.6). The photolyase protein was photoreduced by blue light (440–460 nm, 50 μmol m^−2^ s^−1^) for 30 min before mixing, and then the mixtures were incubated for 15 min and irradiated with the blue light for 2 h. During light illumination, absorbance changes of 260 nm were monitored with a spectrophotometer (UV2550, Shimadzu, Shanghai, China). The enzyme activity was evaluated based on the increase in absorbance. The empty NL was used as a control.

### 4.4. Animal Experiments

Wild-type eight-week-old male Kunming mice (weight 20–22g) were purchased from the Jinan Pengyue Animal Bleeding Co., Ltd. and housed in the Animal Laboratory of Qingdao University of Science and Technology under specific pathogen-free conditions (a 12 h light/dark cycle, temperature of 23 ± 2 ℃, and humidity of 50 ± 10%). Animal experiments were carried out in accordance with Chinese law and the local ethics committee’s regulations on animal welfare. Prior to the animal experiments, the certain quality of freeze-dried CPDNL powders were dissolved with ultrapure distilled water to obtain the CPDNL concentrations of 10 mg/mL and 20 mg/mL. Mice were assigned to five groups (six mice per group) as follows: Norm group (no UVB), UVB group (only UVB), positive VC group (UVB, 0.1 mg/mL vitamin C), L-CPDNL group (UVB, 10.0 mg/mL CPDNL), and H-CPDNL group (UVB, 20.0 mg/mL CPDNL). The hair on the backs of the mice was removed using 8% sodium sulfide prior to UVB exposure, with depilation of an area of 4 cm^2^ (2 cm × 2 cm). The mice were acclimatized for 3 days. Then, the UVB irradiations were performed for 20 min twice per day for 7 days using a UVB lamp of 8 W (wavelength range: 290–320 nm, peak wavelength: 312 nm). The distance from the lamp to the back of mice was 30 cm. The light intensity reached on mice was 0.95 mw/cm^2^, measured with a UVB-radiometer (UV-B, Photoeletric Instrument Factory, Beijing, China). The 100 μL of VC and CPDNL was administered on dorsal skin after UVB exposure. At the end of the experiment, photographs of skin appearance were taken using a Canon camera (DS126521, Canon, Tokyo, Japan). All mice were anesthetized, and the dorsal skin samples were collected according to the later analysis.

### 4.5. Histological Analysis

The dorsal skin tissues were fixed in 10% (*v*/*v*) formalin in PBS for 24 h and embedded into paraffin. The slices were cut at 4 μm and then stained with hematoxylin and eosin (H&E) reagents. The images were observed by a microscope (BX53, Olympus, Tokyo, Japan) equipped with a high-resolution camera (D2X, Nikon, Tokyo, Japan). 

### 4.6. Quantitation of CPDs

Genomic DNA was extracted from skin tissue using a Purelink Genomic DNA mini kit (Thermo Fisher Scientific, Waltham, MA, USA), and CPDs were quantified using the OxiSelect™ UV-Induced DNA Damage ELISA Kit (Cell Biolabs, San Diego, CA, USA), in accordance with the protocol of the manufacturer. Absorbance was measured at 420 nm using an ELISA Microplate Reader (ELX-800, BioTek, Winooski, VT, USA).

### 4.7. Determination of Hydroxyproline Content

The skin tissue was cleaned, homogenized, and hydrolyzed with 6 M hydrochloric acid at 95 ℃ for 5 h. the hydroxyproline concentration was analyzed using hydroxyproline assay kits (A030-3, Nanjing Jiancheng Bioengineering Institute, Nanjing, China), in accordance with the instructions of the manufacturer.

### 4.8. Analysis of Oxidative Stress Level

The skin tissue was gently cleaned with physiological saline (1: 9 *w*/*v*), homogenized, and centrifuged at 4 ℃ at 10,000 rpm for 10 min. The supernatants were collected for ROS and MDA analysis using ROS ELISA kits (MB-6180A, Meibiao Biotechnology, Suzhou, China) and MDA assay kits (A003-1, Nanjing Jiancheng Bioengineering Institute, Nanjing, China), in accordance with the instructions of the manufacturer, respectively. 

### 4.9. RNA Extraction and Quantitative RT-PCR

Total RNA of skin tissue was extracted using TRlzol Reagent and used to synthesize cDNA by TransScript^®^ One-Step gDNA Removal and cDNA Synthesis SuperMix (TransGen Biotech, Beijing, China). Quantitative PCR was performed on Mx3005P QPCR Systems according to TransStart^®^ Top Green qPCR SuperMix (TransGen Biotech, Beijing, China) using the following conditions: one cycle at 95 °C for 30 s, 45 cycles at 95 °C for 5 s, 55–60 °C for 15 s, and then 72 °C for 10 s. The β-actin was used as an internal reference control. Relative gene expression was calculated by relative quantification using comparative Ct (2^−ΔΔCt^) method. The primers sequences are listed in Table 3.

### 4.10. Western Blotting Analysis

Skin tissue was homogenized in RIPA (Radio Immuno Precipitation Assay) lysis buffer, protease inhibitor, and phosphatase inhibitor (Boster, Wuhan, China) to extract the total protein. The protein concentration was determined using BCA protein assay kit. The protein was denatured in gel sample buffer at 100 °C for 5 min, separated by 10% SDS-PAGE, transferred to a polyvinylidene fluoride (PVDF) membrane (EpiZyme, Shanghai, China), and blocked with 5% (*w*/*v*) non-fat dry milk in TBST buffer (Tris-Buffered Saline with Tween-20). The membrane was incubated with the primary antibody against NF-κВ (BA1297-2, Boster, Wuhan, China), TNF-α (BA14901, Boster), IL-6 (BA4339-2, Boster), COX-2 (BA3708, Boster), ERK (GB112238, Servicebio, Wuhan, China), JNK (M02608-3, Boster), P38 (A00176-2, Boster), AP-1 (GB11270, Servicebio), MMP-1 (A00733-1, Boster), and MMP-2 (A00286, Boster) at 4 °C overnight, followed by washing at TBST, and then incubated with goat anti-rabbit HRP (horseradish peroxidase)-IgG (BA1055, Boster) as the secondary antibody for 45 min. After washed with TBST 3 times for 10 min, membrane was incubated in SuperSignal ECL Western substrate (Biosharp, Hefei, China). The protein band was visualized using the Chemiluminescent Imaging System (Tanon-5200SF, Shanghai, China). The intensity was quantified by Image J version 1.8.0 program.

### 4.11. Statistical Analysis

The results were expressed as means ± SEM (*n* = 6). One-way analysis of variance (ANOVA) followed by Tukey’s post hoc test was used to test differences among groups, and *p* < 0.05 was considered statistically significant. GraphPad Prism 8.0.2 and SPSS 18.0 software were used for statistical analysis.

## 5. Conclusions

Overall, the optimal preparation conditions for CPDNL from Antarctic microalgae and their underlying mechanisms in UVB-induced damage mice were revealed. The mean particle size and entrapment efficiency of CPDNL in optimal practice were 105.5 nm and 63.3%, respectively. In UVB-irradiated mice, CPDNL exhibited DNA repair and anti-oxidative, anti-inflammatory, and anti-wrinkling properties. CPDNL reduced CPD photoproducts and ROS and MDA levels against oxidative stress, while decreasing TNF-α, IL-6, and COX-2 via the NF-κB signaling pathways in response to inflammation. Importantly, CPDNL prevented collagen degradation by suppressing MMP-1 and MMP-2 via inhibition of the MAPK/AP-1 signaling pathways. Although CPDNL from Antarctic microalgae have demonstrated substantive efficacy in animal models of UVB damage, their beneficial effects need to be unequivocally proven in human populations. Taken together, our results will provide novel insights into the development and utilization of Antarctic CPD photolyase products, and they may offer an effective therapeutic or preventive strategy to protect against UVB damage, photoaging, and skin diseases.

## Figures and Tables

**Table 1:** Response surface methodology experimental design for CPDNL preparation.

No.	Independent Variables				Y1 (nm)	Y2 (%)
	A	B	C	D		
1	1	0.066	3	12.5	215	60.0
2	4	0.066	3	12.5	110	24.5
3	1	0.2	3	12.5	153	40.0
4	4	0.2	3	12.5	120	20.0
5	2.5	0.133	1	5	200	62.0
6	2.5	0.133	5	5	200	55.0
7	2.5	0.133	1	20	97	49.0
8	2.5	0.133	5	20	116	44.3
9	1	0.133	3	5	170	74.4
10	4	0.133	3	5	81	50.0
11	1	0.133	3	20	117	50.0
12	4	0.133	3	20	58	40.0
13	2.5	0.066	1	12.5	163	74.7
14	2.5	0.2	1	12.5	146	60.0
15	2.5	0.066	5	12.5	213	47.9
16	2.5	0.2	5	12.5	53	42.0
17	1	0.133	1	12.5	155	67.8
18	4	0.133	1	12.5	102	40.0
19	1	0.133	5	12.5	253	78.2
20	4	0.133	5	12.5	150	50.0
21	2.5	0.066	3	5	262	71.0
22	2.5	0.2	3	5	98	65.0
23	2.5	0.066	3	20	140	60.0
24	2.5	0.2	3	20	66	52.0
25	2.5	0.133	3	12.5	99	78.5
26	2.5	0.133	3	12.5	118	72.3
27	2.5	0.133	3	12.5	115	74.0
28	2.5	0.133	3	12.5	89	68.8
29	2.5	0.133	3	12.5	69	60.8

A, ratio of lecithin (g) to cholesterol (g); B, ratio of CPD photolyases (g) to lecithin (g); C, ratio of oil (mL) to water (mL); D, sonication time (min); Y1, mean particle size (nm); Y2, encapsulation efficiency (%).

**Table 2:** Analysis of variance of the quadratic regression equation for particle size (Y1) and encapsulation efficiency (Y2) of CPDNL.

No.	Y1			Y2		
	Mean Square	*F*-Value	*p*-Value	Mean Square	*F*-Value	*p*-Value
Model	5138.53	4.39	0.0045	337.75	2.50	0.0488
A	16,280.33	13.92	0.0022	1773.90	13.13	0.0028
B	18,174.08	15.54	0.0015	291.07	2.15	0.1642
C	1240.33	1.06	0.3205	108.60	0.80	0.3851
D	14,490.75	12.39	0.0034	561.70	4.16	0.0608
AB	1296.00	1.11	0.3103	60.06	0.44	0.5157
AC	625.00	0.53	0.4768	0.04	0.0003	0.9865
AD	225.00	0.19	0.6676	51.84	0.38	0.5455
BC	5112.25	4.37	0.0552	19.36	0.14	0.7107
BD	2025.00	1.73	0.2093	1.00	0.007	0.9327
CD	90.25	0.08	0.7852	1.32	0.01	0.9226
A^2^	2160.41	1.85	0.1956	1329.98	9.85	0.0073
B^2^	4094.70	3.50	0.0823	864.44	6.40	0.0240
C^2^	9739.86	8.33	0.012	150.64	1.12	0.3088
D^2^	454.97	0.39	0.5428	136.91	1.01	0.3311
Residual	1169.27			135.08		
Lack of fit	1475.77	3.66	0.1113	171.54	3.90	0.1006
Pure error	403.00			43.94		

A, ratio of lecithin (g) to cholesterol (g); B, ratio of CPD photolyases (g) to lecithin (g); C, ratio of oil (mL) to water (mL); D, sonication time (min); Y1, mean particle size (nm); Y2, encapsulation efficiency (%).

**Table 3:** Real-time quantitative PCR primer sequences.

Gene		Prime Sequences (5′-3′)
β-actin	Forward	TATGCTCTCCCTCACGCCATCC
	Reverse	GTCACGCACGATTTCCCTCTCAG
TNF-α	Forward	AGATGATCTGAGTGTGAGGGTCTGG
	Reverse	CACCACGCTCTTCTGTCTACTGAAC
COX-2	Forward	CTGGTGCCTGGTCTGATGATGTATG
	Reverse	GGATGCTCCTGCTTGAGTATGTCG
IL-6	Forward	CTTCTTGGGACTGATGCTGGTGAC
	Reverse	TCTGTTGGGAGTGGTATCCTCTGT
NF-κВ	Forward	GGATATGAGGAAGCGGCATGTAGAG
	Reverse	CCTGATACTGGCACTTCGGACAAC
JNK	Forward	CGCCTTATGTGGTGACTCGCTAC
	Reverse	CTCCCATGATGCACCCAACTGAC
ERK	Forward	GCCTTCCAACCTCCTGCTGAAC
	Reverse	CGTACTCTGTCAAGAACCCTGTGTG
P38	Forward	CTGGCTCGGCACACTGATGATG
	Reverse	GCCCACGGACCAAATATCCACTG
AP-1	Forward	CTTCTACGACGATGCCCTCAACG
	Reverse	GCCAGGTTCAAGGTCATGCTCTG
MMP-1	Forward	ACAGTTGACAGGCTCCGAGAAATG
	Reverse	CCACATCAGGCACTCCACATCTTG
MMP-2	Forward	ACCATGCGGAAGCCAAGATGTG
	Reverse	AGGGTCCAGGTCAGGTGTGTAAC

## Data Availability

Not applicable.

## Abbreviations

6-4 PP	pyrimidine-pyrimidone (6-4) photoproducts
AK	actinic keratosis
AP-1	activator protein-1
COX-2	cyclooxygenase-2
CPD	cyclobutane pyrimidine dimer
CPDNL	nanoliposomes of CPD photolyases
ERK	extracellular signal-regulated kinase
JNK	c-Jun-N-terminal kinase
MAPK	mitogen-activated protein kinases
MDA	malondialdehyde
MMP	matrix metalloproteinase
NER	nucleotide excision repair
NF-κB	nuclear factor-κB
NMSC	non-melanoma skin cancer
PBS	phosphate-buffered saline
PDI	polydispersity index
ROS	reactive oxygen species
RSM	response surface methodology
SDS-PAGE	sodium dodecyl sulfate polyacrylamide gel electrophoresis
TNF-α	tumor necrosis factor-α
UVB	ultraviolet-B
XP	xeroderma pigmentosum
